# Unlocking the potential of adeno-associated virus in neuroscience: a brief review

**DOI:** 10.1007/s11033-024-09521-6

**Published:** 2024-04-22

**Authors:** Antea Minetti

**Affiliations:** 1 Neuroscience Institute, National Research Council (CNR), Pisa, Italy; 2 Department of Biology, Unit of Cell and Developmental Biology, University of Pisa, Pisa, Italy

**Keywords:** Adeno-associated virus, Viral vectors, Neuroscience

## Abstract

Adeno-associated virus (AAV) has emerged as a pivotal tool in neuroscience research, owing to its remarkable versatility and efficiency in delivering genetic material to diverse cell types within the nervous system. This mini review aims to underscore the advanced applications of AAV vectors in neuroscience and their profound potential to revolutionize our understanding of brain function and therapeutic interventions for neurological disorders. By providing a concise overview of the latest developments and strategies employing AAV vectors, this review illuminates the transformative role of AAV technology in unraveling the complexities of neural circuits and paving the way for innovative treatments. Through elucidating the multifaceted capabilities of AAV-mediated gene delivery, this review underscores its pivotal role as a cornerstone in contemporary neuroscience research, promising remarkable insights into the intricacies of brain biology and offering new avenues for therapeutic intervention.

AAV was initially discovered as a contaminant of adenovirus stocks in the 1960s. Today, AAVs are currently among the most frequently used viral vectors for gene delivery to the brain due to the lack of cytotoxicity and the long-term expression in neurons [1], moreover they are an attractive candidate for creating viral vectors for gene therapy due to their ability to infect humans [2]. Remarkably, AAV causes a very mild immune response, making it a great candidate for gene therapy with minimal risk of adverse effects. AAV has a linear single strand DNA genome of approximately 4.7 kb in length. Lacking its own polymerase, the AAV genome relies on cellular polymerases for replication. Although AAV wide distribution, its virtually non-existent pathogenicity can be attributed to its inability to replicate on its own. Instead, they necessitate a co-infecting virus capable of replication to initiate a productive infection. In its genome are present only replication and capsid genes for viral replication regulation and capsid structure [3]. During infection cycle AAV enters the cell through receptor-mediated endocytosis and it is transported to the nucleus via clathrin coated vesicle. Once within the nucleus the virion capsid is lost and the viral genome released, where it can either integrate into the host cell genome (in the absence of a co-infected virus) or proceed to lytic cycle (in presence of co-infected virus) [4].

## AAV-mediated gene delivery

Unlike conventional tracers, neurotropic viruses carry information encoded by nucleic acids, they can be genetically engineered for specific desired properties and can be used to introduce foreign genes that can be used to easily detect their presence or, more powerfully, to directly monitor or manipulate neuronal function. Viral-mediated approach enabled space- (targeting a specific brain region) and time-specific approaches (targeting any stage of animal’s life). AAVs are used in neuroscience to efficiently get genetic material into a cell, and even deliver this genetic material to specific cells in an organism.

Depending on experiment aims, researchers can choose the delivery modality of viral particles, either by direct injection into specific anatomic brain regions [5, 6] or via a widespread route [7, 8] for a global viral expression (Table 1). Stereotaxic injection enables infection of a restricted and precise brain area, thereby reducing potential off-target effects, while intravenous and retro-orbital injections (both employing serotypes permeable to the blood-brain barrier) allow uniform, noninvasive gene delivery to the central and peripheral nervous systems [9].

In recent years, significant advancements in neurosurgical techniques have paralleled the progress of AAV-based gene therapy, profoundly impacting the field of neuroscience (reviewed by [10, 11]). Techniques such as convection-enhanced delivery and intrathecal administration have revolutionized the precise targeting and distribution of AAV vectors within the central nervous system [12, 13]. These methods enable widespread transduction of target cells, offering promising therapeutic avenues for conditions like epilepsy [14], neurotransmitter-related disorders [15] and other neurological disorders (reviewed by [16–18]). Additionally, novel vector infusion methods, including focused ultrasound-mediated delivery, enhance the specificity of AAV vector targeting within the CNS, minimizing off-target effects [19, 20]. Ongoing clinical trials and studies have showcased the transformative potential of these advanced neurosurgical approaches in treating a myriad of neurological disorders. These advancements represent a synergistic relationship between AAV vectors and neurosurgical techniques, thereby highlighting their pivotal role in shaping the future of neuroscience research and therapy.

**Table 1:** Viral vectors administration routes: local expression is reached by stereotaxic injection in a precise brain area and global transfection through intravenous, retro-orbital or cerebral spinal fluid injection

	LOCAL	GLOBAL
ADVANTAGES	• Restricted expression to injection site • Time-specific administration at desired developmental stage • Reduced off-target activity respect to global administration • High transgene expression level • Small viral titer and volume	• CNS- or PNS-wide transduction • Uniform viral expression • Sparse or anatomically distributed cell types labeling can be achieved • Permeable to blood-brain barrier • Minimally invasive or non-invasive approach • No need of stereotaxic apparatus or surgical expertise
DISADVANTAGES	• Invasive surgery • Need of stereotaxic apparatus • Tissue damage of the injected area • Expression gradient from injection site • Difficulties in reaching deep brain area	• High volume and viral titer are required • Possible off-target effects • Non-selective approach • Limited by animal body weight

## AAV for controlling brain activity

With the development of cell-specific promoters and enhancers, AAV vectors can target specific cell types within complex brain circuits, allowing researchers to dissect neural circuits with unprecedented precision. AAV vectors boast remarkable specificity, enabling researchers to target distinct cell types and neural circuits with pinpoint accuracy. This precision revolutionizes our understanding of brain function by allowing manipulation at the cellular and circuit levels, unlocking new insights into neural connectivity and correlation to behavior.

The utilization of AAV-mediated delivery for optogenetic and chemogenetic tools offers precise manipulation of neuronal activity, facilitating the exploration of neural circuit dynamics and behavior in both healthy and pathological conditions. These genetic strategies involve the delivery of photoactivatable ion channels named opsins for optogenetics (reviewed by [21–23]) or ligand-gated G-coupled ion channels named designer receptors activated by designer drugs (DREADDs) for chemogenetics (reviewed by [24–26]). In optogenetic applications, the expression of channelrhodopsins (ChR) or halorhodopsins (HR) permits cellular access via light stimulation, enabling the activation or inhibition of neuronal activity within target cells [27, 28]. Conversely, in chemogenetics, the expression of engineered Gq- or Gi-coupled human muscarinic acetylcholine receptors (hM3Dq or hM4Di) enables activation or inhibition via the administration of a specific drug (clozapine N-oxide, CNO) [29]. However, combining these two systems is hindered by the shared drug activation mechanism. To address this limitation, Vardy et al. developed a novel Gi DREADD based on the human κ-opioid receptor, known as KORD, which is activated by a distinct ligand, Salvinorin B (SALB), thus allowing for the implementation of multi-application chemogenetic systems [30]. These technologies allow mapping and dissecting brain circuit in a time- and cell-type specific manner through spatiotemporal expression of chemo- and opto-genetic reporters, which may also be employed in combination due to their different induction methods (light stimulation for optogenetics and drug administration for chemogenetics) [31].

With the advent of optogenetics and chemogenetics, AAV-mediated delivery of light-sensitive and ligand-responsive proteins empowers researchers to modulate neuronal activity with exquisite temporal and spatial resolution. Cre-dependent viral vectors allow efficient gene activation in region- and time-specific manner by delivering the viral vectors to a restricted area at the desired developmental stage. This remarkable control sheds light on the dynamics of neural circuits, offering profound insights into brain function and dysfunction.

## AAV for neuronal tracing

AAV vectors offer high transduction efficiency and long-lasting gene expression in neurons and glial cells, making them invaluable tools for studying gene function and manipulating neural circuits.

Overall, viral tracers can be classified into two categories based on their ability to traverse synapses: non-transsynaptic and transsynaptic. Non-transsynaptic viruses are confined to the neurons they infect and cannot extend across synapses to neighboring neurons. In contrast, transsynaptic viruses have the capacity to cross synapses and propagate to interconnected neurons. Both categories include viruses that are transported either anterogradely or retrogradely along axons. Extensive reviews have provided detailed insights into the diverse properties and applications of these viruses [32, 33]. Notably, among AAV variants, only AAV1 and AAV9 exhibit transsynaptic spreading [34, 35], while others do not.

**Fig. 1:**
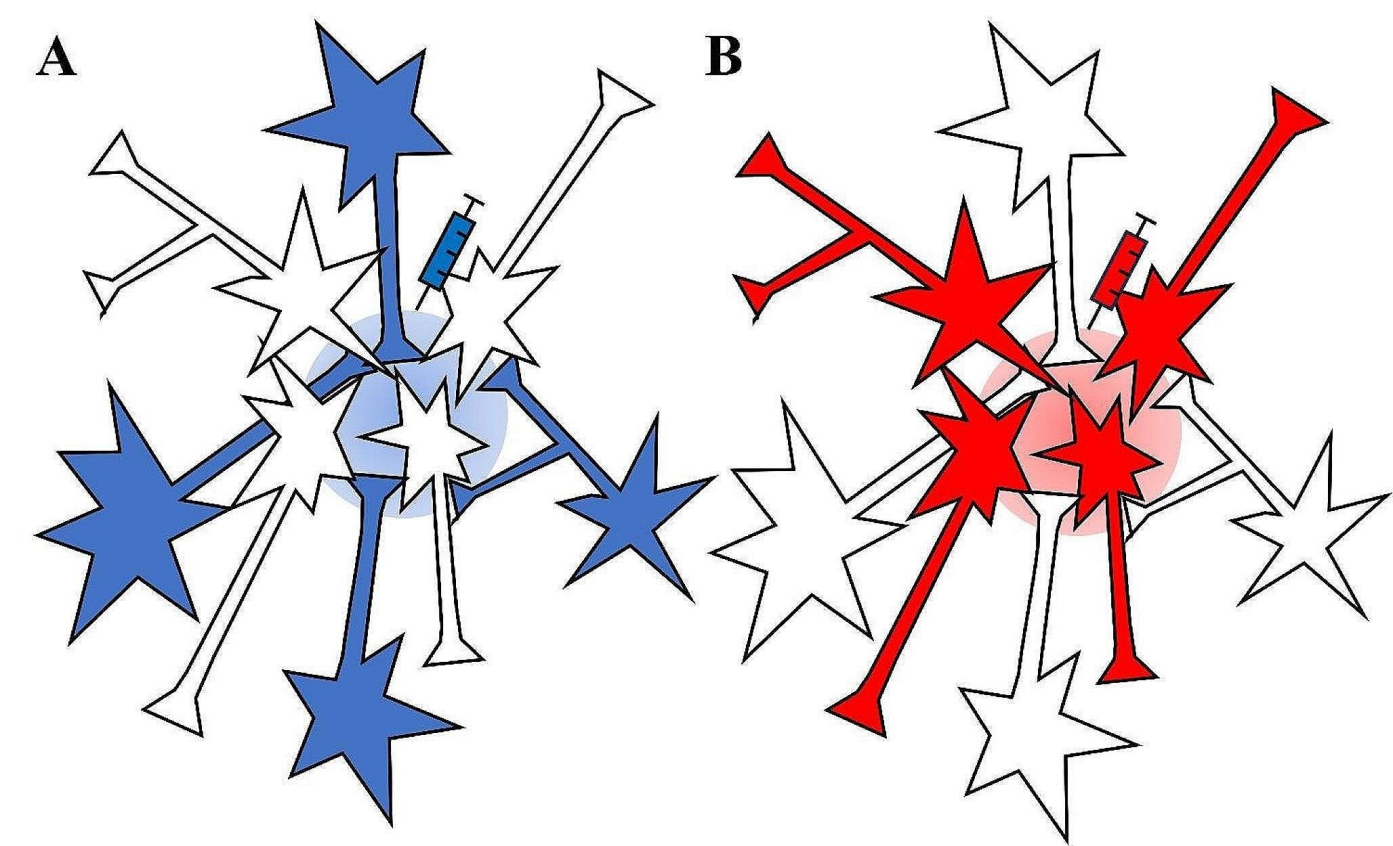
Anterograde and retrograde labeling by viral tracers. Tracers are categorized as anterograde or retrograde based on their direction of travel within neurons, either from or towards the cell body respectively. Anterograde tracers (marked in red) allow the visualization of neurons that receive information from the injection site, since they are absorbed by neuronal cell bodies at the injection site and then travel along the axon to reach terminal processes. Conversely, retrograde tracers (marked in blue) allow to visualize neurons that send projections to the injection site, since they are absorbed by terminals and transported back to the cell body

The direction of spread of each virus type which is essential to identify the neuronal connection to the injection site, especially in tracing experiments. It is fundamental to know the region where the virus is injected, corresponding to the start of infection and the direction of the viral spread and the area of expression, essential to identify and map the neural circuit. For this reason, viral vectors are divided into two categories depending on the directionality of viral transport into the cell respect to the injection site: retrograde and anterograde tracers. Retrograde tracers enter at the level of presynaptic terminal and move retrogradely to cell body of the infected neurons by axonal transport, thus allowing the identification of projecting neurons; vice versa anterograde tracers infect the cell body and are transported to axonal processes, thus allowing the visualization of neurons and their targets [36] (Fig. 1). Most AAV serotypes exhibit anterograde transport properties, as exemplified by one of the latest tools, AAV13, with rigorous anterograde labeling [37]. Conversely, AAV2-retro [38], AAV9-retro [39], AAV-DJ/9 [40] and AAV11 [41] are retrogradely transported.

## AAV engineering and capsid optimization

Numerous strains of AAV have been discovered in nature, categorized into various serotypes based on the unique antigenicity of their capsid proteins. These distinct serotypes exhibit varying tissue tropism, dictating their specificity for infecting different tissues (Table 2). By utilizing different capsid proteins during packaging, AAV vectors can adopt diverse serotypes. Active research is focused on the identification of capsid variants having a superior efficacy profile [42].

To improve and refine AAV tropisms, extensive research has been conducted on the mutational tolerance of AAV capsids. Permissive sites for deliberate or random amino acid substitutions and insertions have been identified through various studies [43, 44]. Additionally, methods for constructing serotype-shuffled chimeric capsids have been developed (for detailed information see the following articles [45–47]).

Numerous engineered capsids with distinct tropisms encompass variants that exhibit: *(i)* confined or broad transduction within the central nervous system (CNS) or peripheral nervous system (PNS); *(ii)* cell-type specificity; or *(iii)* retrograde transport in neurons.

i) Stereotaxic injection facilitates infection within a confined and specific region of the brain, minimizing the risk of off-target effects. In contrast, intravenous and retro-orbital injections, utilizing serotypes capable of crossing the blood-brain barrier, offer consistent and non-invasive gene delivery to both the central and peripheral nervous systems [9]. Moreover, numerous serotypes exhibit broad neuronal and glial transduction within the brain following direct intraparenchymal injection [48].
ii) Cell-type specific infection can be accomplished through the integration of AAV delivery with recombinase systems or gene expression driven by tissue-specific promoters. Cre-dependent viral vectors enable precise gene activation in both spatial and temporal manners by directing the viral vectors to a delimited area during the desired developmental stage [5, 8]. The collaboration between viral vectors and the Cre/*lox* system has vastly expanded their utility, capitalizing on the adaptability of recombinase-based technology and the rapidity and adaptability of viral injections. AAV can carry the gene of interest under the control of a constitutive promoter like CMV or EF1a promoter [49], or it can be selectively expressed in a more defined subset of cells using a tissue-specific promoter to explore gene profiles and functions within various subpopulations [50, 51].
iii) Targeting the soma of projection neurons becomes crucial for selectively manipulating neurons that terminate at specific sites. Achieving this specificity involves delivering an AAV engineered for retrograde transduction, such as AAV2-retro [38], AAV9-retro [39], AAV-DJ/9 [40], and AAV11 [41] to the projection sites. Consequently, only the cell bodies of neurons terminating in that area will be effectively targeted.

Ongoing efforts to engineer AAV capsids for enhanced tropism, transduction efficiency, and reduced immunogenicity are expanding the utility of AAV vectors in neuroscience research and gene therapy.

**Table 2:** Different AAV serotypes for tissue specificity

TISSUE	AAV SEROTYPES
Brain	AAV1, AAV2, AAV5, AAV8, AAV9, AAV11, AAV13, AAV-PHP.eB, AAV2-retro, AAV9-retro AAV-DJ/9
Retina	AAV1, AAV2, AAV4, AAV5, AAV8, AAV9
Spinal nerves	AAV2-retro, AAV-PHP.S

## Limitations

In this mini-review, we’ve explored the advanced applications of adeno-associated virus (AAV) vectors in neuroscience. While AAV vectors offer several advantages, including long-term gene expression, and low immunogenicity, it is important to acknowledge certain limitations, particularly regarding transduction efficiency. Despite their widespread use and efficacy, AAV vectors may not achieve the same level of transduction efficiency as some other viral vectors. For instance, comparative studies have shown that AAV vectors typically require higher multiplicity of infection (MOI) compared to certain other viral vectors, such as lentiviral vectors, to achieve similar levels of transduction [52, 53]. Several factors contribute to the limitations of AAV transduction efficiency. These include the natural tropism of AAV serotypes [54, 55], which may not match the target cell type, as well as the presence of pre-existing neutralizing antibodies in the host that can inhibit vector entry [56–58]. Additionally, the relatively small packaging capacity of AAV vectors restricts the size of the transgene that can be delivered, limiting their utility for certain applications requiring larger genetic payloads. Researchers should carefully consider these limitations when choosing vector systems. While AAV’s safety and targeted delivery are advantageous, vectors like herpes simplex virus (HSV) or lentivirus may offer higher transduction efficiency due to their unique characteristics. While AAV remains a valuable tool, its limitations in transduction efficiency must be taken into account.

## Conclusions

In conclusion, AAV vectors have revolutionized neuroscience research by enabling precise manipulation of neural circuits and offering promising therapeutic strategies for treating neurological disorders. Continued advancements in vector design and delivery techniques will further enhance the utility and efficacy of AAV-based approaches in unraveling the complexities of the brain and developing novel treatments for neurological diseases.

## Data Availability

No datasets were generated or analysed during the current study.
