# Correction: Unlocking the potential of adeno-associated virus in neuroscience: a brief review

**DOI:** 10.1007/s11033-024-09671-7

**Published:** 2024-06-05

**Authors:** Antea Minetti

**Affiliations:** 1 Neuroscience Institute, National Research Council (CNR), Pisa, Italy; 2 Department of Biology, Unit of Cell and Developmental Biology, University of Pisa, Pisa, Italy; 3 Functional Neuroimaging Laboratory, Istituto Italiano di Tecnologia, Center for Neuroscience and Cognitive Systems @UniTn, Rovereto, Italy

**Correction to: Molecular Biology Reports (2024) 51:563**

10.1007/s11033-024-09521-6

In this article the affiliation ‘Functional Neuroimaging Laboratory, Istituto Italiano di Tecnologia, Center for Neuroscience and Cognitive Systems @UniTn, Rovereto, Italy’ for the author Antea Minetti was missing.

